# The Ocular Microbiome and Alzheimer’s Disease: Emerging Insights

**DOI:** 10.3390/cells15171590

**Published:** 2026-09-01

**Authors:** Shyam Kumar Mishra, Jerome Ozkan, Feifei Su, Woojin Scott Kim, Mark Willcox, YuHong Fu

**Affiliations:** 1 School of Optometry and Vision Science, University of New South Wales, Sydney, NSW 2052, Australia; s.mishrabaishnab@unsw.edu.au (S.K.M.); j.ozkan@unsw.edu.au (J.O.); 2 School of Medical Sciences, The University of Sydney, Sydney, NSW 2006, Australia; feifei.su@sydney.edu.au (F.S.); woojin.kim@sydney.edu.au (W.S.K.); 3 Brain and Mind Centre, The University of Sydney, Camperdown, NSW 2050, Australia

**Keywords:** Alzheimer’s disease, ocular microbiome, neurodegeneration, dysbiosis, gut–brain connection, low-biomass microbiome, retina, neuroinflammation

## Abstract

**Highlights:**

This review highlights the growing potential of microbiome research to broaden understanding of Alzheimer’s disease. While the gut and oral microbiomes currently provide the strongest evidence, the ocular microbiome represents an exciting emerging area, supported by the close biological relationship between the eye and brain and offering potential opportunities for novel biomarkers and targeted therapeutic strategies.

**What are the main findings?**

- The gut and oral microbiomes currently provide the strongest evidence linking microbial dysbiosis to Alzheimer’s disease, while the ocular microbiome has emerged as a biologically plausible but largely unexplored component of the eye–brain connection.
- The ocular surface shares anatomical and functional connections with the central nervous system, and overlaps between ocular microbial taxa and those reported in some Alzheimer’s disease brain studies provide a rationale for investigating a potential ocular-brain microbial interface.

**What are the implications of the main findings?**

- The ocular microbiome represents a novel frontier in Alzheimer’s disease research that may provide unique insights into neuroinflammation, retinal neurodegeneration, and brain health through a readily accessible tissue interface.
- Integrating ocular microbiome profiling with retinal imaging, tear biomarkers, and spatial biology approaches could enable the discovery of new biomarkers and therapeutic targets for Alzheimer’s disease, provided that future studies establish causal mechanisms and address methodological challenges in low-biomass microbiome research.

**Abstract:**

Alzheimer’s disease (AD) is a progressive neurodegenerative disorder characterized by amyloid-β plaques, neurofibrillary tau tangles, neuroinflammation, and progressive cognitive decline. Beyond these classical pathological features, emerging evidence implicates microbial dysbiosis as a contributing factor, with the gut and oral microbiomes currently providing the strongest evidence for microbiome AD associations. In contrast, the ocular microbiome represents a biologically plausible but largely unexplored candidate whose potential contribution to AD pathogenesis remains hypothetical and requires rigorous investigation. The ocular surface shares embryological, anatomical, and functional connections with the central nervous system, and the retina has emerged as a non-invasive window into neurodegenerative brain changes. Bacterial taxa detected in ocular specimens, including *Cutibacterium acnes* and *Acinetobacter johnsonii*, overlap with those reported in some studies of AD brain tissue, prompting speculation about an ocular–brain microbial interface. However, this overlap does not establish microbial trafficking, and no direct evidence currently supports the proposition that ocular microorganisms translocate to, colonize, or contribute causally to AD brain pathology. This review critically appraises the existing evidence, graded by methodological rigor and evidence strength, across gut, oral, nasal, ear, and ocular microbiome compartments. We address the substantial methodological challenges inherent in low-biomass microbiome research, emphasize the importance of distinguishing contamination artifacts from biological signals, and delineate the evidence gaps that separate association from causation. We conclude by proposing a research framework that places the ocular microbiome as an emerging hypothesis warranting experimental validation, rather than an established contributor to AD.

## 1. Introduction

More than 57 million people were estimated to live with dementia worldwide in 2021, a figure projected to rise to 139 million by 2050, with medical costs forecast to exceed one trillion USD [1]. Alzheimer’s disease (AD) accounts for 60–80% of dementia cases and is characterized by progressive impairments in memory, cognition, reasoning, and behavior, typically in older populations [2]. Crucially, pathological changes, including amyloid-β (Aβ) deposition and tau hyperphosphorylation, begin to accumulate in the brain years to decades before the onset of overt clinical symptoms, underscoring the importance of early biomarker identification and mechanistic understanding [3]. The classical amyloid cascade hypothesis, while remaining influential, has been challenged by the limited cognitive benefits of Aβ-targeting immunotherapies and the failure of numerous clinical trials targeting downstream pathological features [4,5]. These limitations have prompted exploration of additional and intersecting pathogenic mechanisms, including neuroinflammation, vascular dysfunction, mitochondrial failure, glymphatic clearance impairment, and, most recently, microbial dysbiosis. The ‘microbiota hypothesis’ of AD proposes that perturbations in microbial communities at various body sites may contribute to disease pathogenesis through systemic inflammation, disruption of host barriers, and direct or indirect effects on central nervous system (CNS) neuroinflammation and protein aggregation [6].

The existence of a resident brain microbiome in healthy individuals remains controversial and should not be assumed from current evidence. The detection of microbial DNA in brain tissue does not constitute evidence of viable colonization, active infection, or causal involvement in neurodegeneration. The brain has traditionally been considered a sterile organ protected by the blood–brain barrier (BBB), and the controversy surrounding analogous claims for the placental microbiome provides a cautionary precedent for how technical artifacts can generate misleading biological conclusions in low-biomass settings [7]. These methodological concerns must be addressed before any site-specific microbiome can be credibly implicated in AD pathogenesis.

Among the non-gut microbiome compartments now under investigation, the ocular surface has attracted growing interest [8,9,10]. The eye shares close anatomical and developmental relationships with the brain. The retina is a direct extension of the CNS and ocular structural and functional changes have emerged as sensitive biomarkers of early neurodegeneration [11,12]. Several bacterial taxa found on the healthy ocular surface overlap with those reportedly detected in some AD brain studies [13,14,15,16], and conditions such as dry eye disease, age-related macular degeneration (AMD), and glaucoma share epidemiological, pathological, and molecular features with AD [17,18,19,20]. However, whether the ocular microbiome contributes actively to AD pathogenesis, or whether these overlaps reflect shared age-related ecological and immunological changes, remains unresolved.

**Figure 1:**
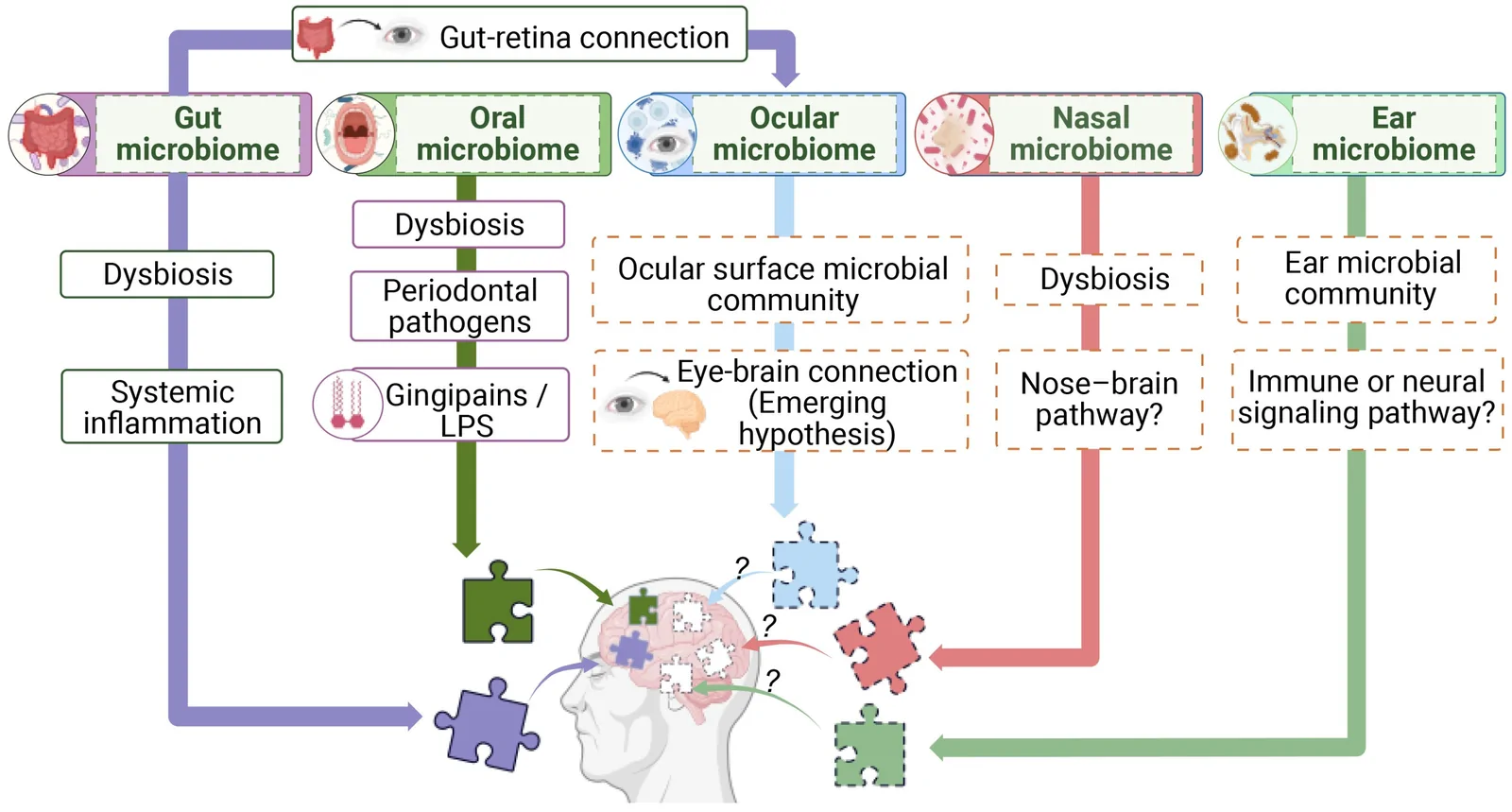
Proposed mechanisms linking body-site microbiomes to Alzheimer’s disease (AD). The gut, oral, ocular, nasal, and ear microbiomes may contribute to neurodegeneration through distinct but interconnected pathways. Gut dysbiosis may promote systemic inflammation, while oral pathogens and their virulence factors, including gingipains and lipopolysaccharide (LPS), may induce neuroinflammatory responses. The ocular microbiome may influence brain health through alterations in the ocular surface microbial community and the putative eye–brain connection. Emerging evidence suggests that the nasal and ear microbiomes may also modulate neurological processes through microbial, immune, and inflammatory signaling pathways. Collectively, these microbiome-derived factors may converge to influence brain pathology associated with AD. Question marks indicate mechanisms that remain incompletely understood. Figure created using BioRender.

This review aims to critically summarize the current state of evidence linking multiple microbiome compartments to AD, with explicit acknowledgment of evidence strength; place the ocular microbiome within this landscape as an emerging but unproven hypothesis; address the methodological challenges inherent to low-biomass microbiome research; and propose a rigorous research framework to determine whether ocular dysbiosis is a driver, consequence, or bystander of AD-related neurodegeneration (Figure 1). A comprehensive literature search was conducted using PubMed, Scopus, and Google Scholar with combinations of terms, including “Alzheimer”, “dementia”, “neurodegenerative”, “gut”, “oral”, “periodontal”, “olfactory”, “nasal”, “ear”, “ocular”, “eye”, “microbiome”, “microbiota” using Boolean operators as appropriate. No restriction was placed on the publication period. Priority was given to peer-reviewed English-language human studies, while relevant animal studies were included to provide mechanistic support. Reference lists of selected articles were also screened to identify additional relevant studies. Preprints and articles outside the scope of microbiota/microbiome involvement in AD were excluded.

## 2. Pathogenesis of Alzheimer’s Disease: A Framework for Microbial Integration

The pathogenesis of AD is multifactorial [21] and involves the convergence of genetic risk, aging, vascular dysfunction, immunological dysregulation, and increasingly recognized microbial influences. Table 1 summarizes the principal hypotheses of AD pathogenesis with explicit commentary on their relevance to microbiome-related mechanisms. Understanding this broader mechanistic landscape is an essential context for evaluating the biological plausibility of any proposed microbiome AD link. A unifying thread across these hypotheses is the role of chronic neuroinflammation, driven at least in part by the accumulation of misfolded proteins and, potentially, by persistent exposure to microbial components. The antimicrobial peptide hypothesis proposes that Aβ itself functions as an innate immune effector against pathogens, and that chronic microbial stimulation leads to its maladaptive accumulation [22]. This framework accommodates microbial contributions from multiple anatomical sites without requiring direct brain colonization, a distinction of critical importance when evaluating the evidence. Of particular relevance to the ocular compartment, glymphatic failure has emerged as an integrating mechanism: impaired sleep-driven interstitial fluid clearance allows Aβ and other toxic proteins to accumulate in both the brain and the retina, and elevated intraocular pressure compresses the optic nerve sheath, further restricting periocular glymphatic outflow [23]. This provides a mechanistic link between ocular physiology and CNS protein homeostasis that is independent of, but potentially compounded by, microbial influences.

Retinal Aβ accumulation is likely multifactorial, reflecting both increased local production and impaired clearance. Retinal neurons and glial cells possess amyloid processing protein (APP)-processing machinery, while oxidative stress, mitochondrial dysfunction, metabolic impairment, and chronic inflammation may promote amyloidogenic processing and increase Aβ production. Impaired clearance may also contribute through age-related dysfunction of retinal and perioptic fluid drainage pathways, altered vascular function, and disruption of the blood–retinal barrier (BRB). Chronic neuroinflammation may further exacerbate Aβ accumulation, as prolonged microglial activation can impair Aβ clearance while promoting oxidative stress and cytokine release. Astrocytes and Müller glia may similarly contribute to this inflammatory environment. Microbial products or persistent infection may provide an additional stimulus by activating Toll-like receptors and the NLRP3 inflammasome, leading to glial activation and cellular stress that could promote Aβ accumulation and impair its clearance. These effects may arise from systemic or local infection without requiring direct ocular-to-brain microbial trafficking. Thus, retinal Aβ accumulation may result from the convergence of altered APP processing, impaired clearance, vascular dysfunction, oxidative stress, and chronic innate immune activation.

Given that amyloidogenic Aβ42 exhibits direct antimicrobial and agglutinating activity against bacteria and fungi, and that retinal Aβ deposition is well documented in AD, an unexplored possibility is that locally produced Aβ at the ocular surface or retina could itself reshape the ocular microbiome, potentially disrupting the balance between commensal and pathogenic taxa through non-selective antimicrobial activity, in parallel to its proposed role in the gut and CNS. This would establish a feed-forward loop in which dysbiosis-driven inflammation induces local Aβ production as an innate immune response, while antimicrobial activity of Aβ itself further reshapes microbial community structure, a hypothesis directly and worthy of being testable through combined ocular Aβ immunostaining and 16S microbiome profiling in aged and AD-model eyes.

Genetic susceptibility should also be considered when evaluating retinal microbial and inflammatory mechanisms in AD. APOE4, the strongest common genetic risk factor for late-onset AD, influences lipid transport, Aβ metabolism, vascular integrity, and innate immune responses. These functions may also affect the retinal response to infection and inflammation. Recent evidence from Gaire et al. identified *Chlamydia pneumoniae* inclusions in post-mortem human retinas and demonstrated a higher pathogen burden in AD that was associated with APOEε4 status, increasing disease severity and cognitive impairment [24]. Retinal *C. pneumoniae* burden was associated with Aβ42 accumulation and activation of the NLRP3 inflammasome, while experimental infection promoted Aβ production, neuroinflammation, and neurotoxicity. These findings raise the possibility that APOE4 may increase susceptibility to infection-associated inflammatory pathology or impair the clearance of pathogen-associated and amyloid-related material. However, the available evidence remains associative, and the mechanisms through which APOE4 influences retinal host–microbe interactions require further investigation.

**Table 1:** Proposed hypotheses of Alzheimer’s disease pathogenesis and their relevance to microbiome and ocular research.

Hypothesis	Core Mechanism	Relevance to Microbial/Ocular Link	References
Amyloid cascade	APP cleavage by β/γ-secretases → Aβ oligomers → plaque formation, neuroinflammation, synaptic loss. Aβ plaques found in retina.	Microbial lipopolysaccharide (LPS) and virulence factors promote Aβ aggregation; retinal Aβ detectable non-invasively.	[25,26,27,28,29,30,31,32]
Tau/Neurofibrillary tangles	Hyperphosphorylated tau → neurofibrillary tangles → axonal transport failure, neuronal death. Tau inclusions found in post-mortem retinas.	Periodontal gingipains directly modify tau; retinal tau pathology mirrors brain burden.	[11,33,34,35]
Microbiota–gut–brain connection	Gut dysbiosis → impaired gut barrier → systemic LPS → BBB disruption → neuroinflammation → amyloidosis.	Strongest current microbial evidence for AD; provides mechanistic framework for other microbiome niches.	[36]
Microbial infection/innate immunity	Chronic infection (HSV-1, *P. gingivalis*) → Aβ as antimicrobial peptide response → maladaptive accumulation.	Oral, nasal and ocular microbes are plausible chronic infection sources.	[37,38]
Endotoxin (LPS)	Gram-negative bacterial LPS → microglial activation → synapse and neuron phagocytosis.	LPS detected in AD brains; oral and ocular Gram-negative dysbiosis may contribute.	[39,40]
Neuroinflammation/Microglia	Chronic microglial activation → impaired Aβ clearance → paradoxical phagocytosis of healthy synapses → neurodegeneration.	Microbial products (e.g., LPS and gingipains) activate microglia; retinal microglia show same dysregulation.	[37,38,39,40]
Vascular-immune	Impaired cerebral blood flow → beta site amyloid precursor protein cleaving enzyme 1 upregulation → Aβ production; BBB breakdown precedes plaque deposition.	Ocular vascular changes (Optical Coherence Tomography Angiography) reflect cerebrovascular pathology; BRB parallels BBB disruption.	[41,42]
Glymphatic failure	Impaired sleep-driven glymphatic clearance → Aβ/α-syn accumulation; Intraocular pressure elevation compresses optic nerve sheath → impaired ocular waste clearance.	Ocular nerve sheath diameter (Optical Coherence Tomography) is non-invasive glymphatic proxy; sleep disruption common in AD.	[23,43,44,45]
Mitochondrial dysfunction	Energy deficits, oxidative stress, impaired calcium homeostasis precede Aβ/tau pathology.	Ocular ALDH enzymes (ALDH3A1) protect against mitochondrial oxidative aldehyde accumulation.	[46,47]
Cholinergic	Loss of cholinergic neurons, reduced ACh → cognitive decline; AChE promotes Aβ production.	Tear biomarkers and pupillary responses reflect cholinergic status.	[48]

## 3. The Microbiome-Brain Connection in Alzheimer’s Disease

The BBB normally protects the brain from most circulating microorganisms. In AD, BBB disruption is increasingly recognized as an early pathological feature, occurring prior to overt Aβ and tau accumulation and providing a potential route of entry for microbial products and, in principle, viable microorganisms [49,50]. Through this compromised barrier, microbial components, LPS, gingipains, and other bacteria-derived proteins may access the CNS and trigger neuroinflammatory cascades, microglial activation, and downstream amyloidogenic responses. Importantly, microbial contributions to AD do not require direct brain colonization. Multiple indirect mechanisms have been proposed, including systemic inflammatory signaling following gut barrier breakdown, vagus nerve-mediated transmission of microbial signals from the gut, receptor-mediated transcytosis of microbial products across the BBB, and Trojan horse mechanisms whereby infected immune cells traffic microbial components into the CNS [9,51]. Antibiotics have been shown to reduce amyloid plaques in rodent models and, in some clinical observations, to slow cognitive decline, lending indirect support to bacterial involvement without specifying the source microbiome [52].

A comprehensive summary of microbial species reported in association with AD, together with the methods used for their detection and the evidence basis for each association, is presented in Table 2. Associations are graded according to evidence strength, from direct brain detection with rigorous controls to bioinformatic inference with no experimental validation.

## 4. The Gut Microbiome in Alzheimer’s Disease: The Strongest Current Evidence

The gut microbiota constitutes the most extensively studied and currently best-evidenced microbiome compartment in relation to AD. The human gut harbors an estimated 3.8 × 10^13^ bacteria, representing over 3000 species across fewer than 130 genera, and is dominated by the phyla *Bacteroidota*, *Bacillota*, *Actinomycetota*, *Pseudomonadota*, *Verrucomicrobiota*, and *Fusobacteria* [70,71]. This community shifts with advancing age, with older populations displaying altered *Bacillota*:*Bacteroidota* ratios and increased *Pseudomonadota*, changes that are increasingly associated with systemic inflammation and metabolic dysfunction [72]. Specific gut microbial signatures have been associated with AD pathology in both human cohort studies and animal models. Pro-inflammatory taxa, including *Escherichia/Shigella*, are increased, while anti-inflammatory organisms such as *Eubacterium rectale* and *Bifidobacterium breve* are reduced in patients with amyloidosis [59,73]. These dysbiotic shifts are proposed to impair gut barrier integrity, increase circulating LPS levels, and promote systemic inflammation that ultimately crosses the BBB, exacerbating neuroinflammation and amyloid aggregation. Recent large-scale analyses confirm that both compositional and functional gut microbiome features are associated with established AD biomarkers, including cerebrospinal fluid (CSF) Aβ and phosphorylated tau [74].

A mechanistically important demonstration of gut–retina connectivity was provided by Peng et al. [75], who showed that compromised colonic epithelial barrier integrity enabled bacterial translocation from the gut to the retina in a mouse model, causing secondary retinal degeneration. This gut–retina connection provides a plausible indirect route through which gut dysbiosis may influence ocular and CNS health. Fecal microbiota transfer studies further show that aged microbiota can induce neuroinflammatory and ocular inflammatory changes in young mice [76]. Together, these findings suggest that microbial dysbiosis may affect the retina and brain simultaneously without requiring direct ocular-to-brain microbial trafficking. Critically, evidence for the gut microbiome in AD, while the most advanced, remains largely associative. Longitudinal cohort data with temporally resolved microbiome profiling, causal inference analyses, and intervention trials are required before any specific gut taxon can be established as a driver rather than a correlate of AD pathology.

## 5. The Oral Microbiome

The oral cavity hosts at least 700 bacterial species across phyla, including *Actinomycetota*, *Bacteroidota*, *Bacillota*, *Fusobacteriota*, and *Spirochaetota*, which are maintained in health by a stable host-microbial balance within the periodontal pocket [77,78]. Disruption of this balance leads to periodontal diseases, such as gingivitis and periodontitis, affecting over 50% of the older population [78,79,80], and provides a well-characterized route for systemic dissemination of microbes and microbial products via the inflamed gingival vasculature. The evidence linking oral dysbiosis to AD is substantially stronger than evidence for any other non-gut microbiome compartment. *Porphyromonas gingivalis*, the principal periodontal pathogen, has been detected directly in AD brain tissue by immunofluorescence and immunoblotting [55]. Its virulence factors, gingipain cysteine proteases, have been shown to degrade innate immune effectors, including apolipoprotein E and the antimicrobial peptide LL-37, directly modify tau protein, promote Aβ aggregation, and disrupt microglial function [37,38]. The passive translocation of *P. gingivalis* to the brain via the bloodstream has been demonstrated to trigger neuroinflammation and neurofibrillary tangle formation in animal models [81].

Epidemiological studies consistently show that periodontal disease is associated with increased AD risk, and serum antibodies to periodontal pathogens, including *P. gingivalis*, *Aggregatibacter actinomycetemcomitans*, *Fusobacterium nucleatum*, and *Treponema denticola*, are elevated in AD patients compared with controls [65]. An oral-derived microbiome has been identified as predominant in AD brains in some sequencing studies, and loss of commensal protective species such as *Streptococcus salivarius* and *Neisseria* spp. may reduce anti-inflammatory protection, exacerbating neuronal damage [82].

It is important to acknowledge that evidence from the oral microbiome for AD, while more developed than ocular evidence, still faces significant methodological limitations. The proposed mechanistic pathways from the periodontal pocket to the bloodstream to the brain involve multiple steps, each requiring experimental verification, and human prospective intervention studies demonstrating that periodontal treatment reduces AD risk or biomarker burden remain limited.

## 6. Nasal, Olfactory, and Ear Microbiomes: Mechanistic Plausibility

### 6.1. Nasal and Olfactory Microbiome

The nasal microbiota undergoes progressive age-related shifts, becoming increasingly dominated by *Staphylococcus*, *Streptococcus*, *Veillonella*, *Cutibacterium*, and *Corynebacterium* in middle age, with further changes in elderly populations [83,84]. Olfactory dysfunction (hyposmia and anosmia) is strongly linked to early AD and is considered a prodromal biomarker, preceding cognitive decline by years in some cohorts [85,86]. Recent evidence has identified 18 nasal bacterial genera associated with olfactory function, with specific biotypes, *Corynebacterium*-dominated profiles, associated with lower prevalence of mild cognitive impairment compared with *Dolosigranulum*- or *Moraxella*-dominated profiles [87]. *C. pneumoniae* has been detected in AD brains using multiple methods, and intranasal inoculation of mice with *C. pneumoniae* induces cerebral Aβ plaques that resemble AD neuropathology [88]. The olfactory nerve provides a direct anatomical route from the nasal cavity to the brain, bypassing the BBB, making the nasal-olfactory pathway a biologically plausible entry route for pathogens in AD [89]. Despite this plausibility, a direct causal link between the nasal microbiome and AD has not been demonstrated. Evidence remains largely observational, and the confounding effects of age-related immune senescence and pre-existing neurodegeneration on nasal microbiome composition remain uncontrolled in existing studies.

### 6.2. Ear Microbiome

The ear represents the least-evidenced microbiome compartment in relation to AD. The adult ear core microbiome comprises *Cutibacterium acnes* alongside *Staphylococcus capitis*, *S. caprae*, and the fungi *Malassezia arunalokei*, *M. globosa*, and *M. restricta* [90]. Some fungi detected in the ear, including *Candida* spp. and *Malassezia* sp., have been reported in brain samples from AD patients, but this overlap is highly circumstantial and is equally attributable to common environmental sources, age-related microbiome convergence, or contamination. No mechanistic pathway linking ear microbiota to AD pathogenesis has been proposed or investigated.

## 7. The Ocular Microbiome/Microbiota and Alzheimer’s Disease

### 7.1. The Ocular Surface Microbiota in Health

The ocular surface is continuously exposed to the external environment and harbors a relatively sparse but reproducible community of microorganisms. Dominant phyla include *Pseudomonadota*, *Bacillota*, *Actinomycetota*, and *Bacteroidota*, with the most prevalent genera including *Acinetobacter*, *Corynebacterium*, *Staphylococcus*, *Cutibacterium*, and *Streptococcus* on the conjunctival surface, and *Corynebacterium*, *Cutibacterium*, and *Staphylococcus* predominating at the lid margin [91,92,93]. Several conserved genera, including *Staphylococcus*, *Corynebacterium*, *Streptococcus*, and *Cutibacterium*, are consistently detected across individuals, supporting the existence of a reproducibly detected core taxa, though individual-specific profiles predominate over a shared population-level community [94,95]. The ocular microbiota varies with age, with older individuals showing relatively higher abundances of *Pseudomonadota* and *Streptococcus*, and age-related shifts in microbial community structure that may accompany changes in lacrimal function, immune senescence, and altered ocular surface physiology [96].

### 7.2. Ocular Pathology, AD-like Features, and the Eye–Brain Relationship

The eye is among the most anatomically and developmentally proximate organs to the brain. The retina is a direct extension of the CNS, sharing embryological origin and molecular architecture with cortical tissue [12,97]. Aβ plaques and hyperphosphorylated tau are detectable in the post-mortem retinas of AD patients, and retinal thinning, quantifiable non-invasively by optical coherence tomography (OCT), has emerged as a sensitive biomarker of AD neurodegeneration [11,32]. Mendelian randomization studies have demonstrated bidirectional genetic and causal relationships between retinal structural traits and AD, suggesting that retinal and cerebral neurodegenerative changes may mutually influence each other during disease progression [98].

Ocular diseases with AD-like pathological features include AMD (Aβ deposition in drusen and complement dysregulation), glaucoma (retinal ganglion cell neurodegeneration and impaired axonal transport), and diabetic retinopathy (vascular dysfunction and early neuroretinal degeneration). Both dry eye disease and AD preferentially affect older females, share overlapping autonomic dysfunction, and may both reflect glymphatic and lipid metabolic failure [99,100]. These epidemiological and pathological parallels do not establish a causal ocular microbiome–AD relationship but provide biological context for why the ocular compartment warrants investigation. The orbitofrontal cortex, which sits directly above the bony orbits, is among the first brain regions to accumulate Aβ plaques, and the eye–brain connection represents a close anatomical and functional interplay whereby visual dysfunction can potentiate structural and functional brain changes [29,101]. This proximity, combined with shared retinal and cortical Aβ and tau pathology, places the eye as a potentially important site in the AD pathological cascade, though whether the ocular surface microbiome participates in this relationship remains a separate, unresolved question.

### 7.3. Ocular Microbiome Taxa Overlapping with AD Brain Findings

The Alzheimer’s Pathobiome Initiative (AlzPI) study identified *Cutibacterium acnes* and *Acinetobacter johnsonii* as the most prevalent bacterial taxa associated with AD brain tissue using full-length 16S rRNA microbiome analysis with rigorous contamination controls [13]. Both species are commonly detected in normal ocular specimens: *C. acnes* as a skin and lid margin commensal, and *Acinetobacter* species as environmental Gram-negative organisms [14,15]. This overlap between AD-associated brain taxa and normal ocular residents has been interpreted by some as suggestive of an ocular–brain microbial interface. However, this interpretation should be approached with considerable caution. The alternative explanations are equally or more plausible: (1) both the ocular surface and brain tissue are prone to environmental contamination with ubiquitous skin commensals during sampling and processing; (2) these organisms are among the most common bacteria on human skin surfaces generally, and their co-detection in eye and brain samples may reflect a shared environmental source rather than biological trafficking; (3) ageing produces ecological convergence across multiple body sites, leading to similar microbiome shifts at unrelated locations; and (4) methodological factors specific to low-biomass sequencing amplify the signals of contaminating skin organisms at both sampling sites. However, the observation of differential microbial profiles between AD and control samples suggests that contamination alone may not fully account for the reported findings, though its contribution cannot be ruled out.

Experimental work in Wistar rats has demonstrated that intracerebroventricular injection of *C. acnes* produces AD-like hippocampal pathology, including Aβ accumulation, TLR2/NF-κB/NLRP3 inflammasome activation, reactive gliosis, and oxidative stress markers [102]. This experimental model, however, does not demonstrate that ocular *C. acnes* traffic to the brain under physiological or disease conditions in humans. At present, there is no direct evidence that ocular microorganisms colonize the AD brain. There is also no evidence that ocular dysbiosis precedes AD onset or that manipulating the ocular microbiome alters AD pathology in experimental models. The proposed ocular–brain microbial connection should therefore remain a hypothesis requiring rigorous experimental validation. In summary, any proposed ocular–brain microbial linkage should currently be regarded as a hypothesis requiring experimental validation through rigorous investigation. The available evidence remains preliminary to support the existence of a functional ocular microbiome–AD connection.

### 7.4. Gut–Retina Connectivity as an Indirect Pathway

A potentially more tractable route by which the microbiome may affect both ocular and brain health simultaneously involves the gut. Parker et al. [76] demonstrated that fecal microbiota transplantation from aged into young mice promoted neuroinflammation and altered inflammatory protein expression in ocular tissues, including enhanced microglial activation in the brain and increased intestinal permeability, changes that were partially reversed by transplanting young microbiota into aged recipients. In a separate study, Peng et al. [75] showed that bacterial translocation from the gut to the retina via a compromised colonic-retinal barrier drove secondary retinal degeneration in a murine model. These findings suggest that the gut microbiome may influence both retinal and CNS inflammatory states via systemic routes, providing a mechanistic framework that does not require direct involvement of the ocular surface microbiome. The emphasis on the gut–retina connection reflects the comparatively stronger experimental evidence for this pathway rather than the exclusion of other microbiome niches. Although oral and nasal dysbiosis have plausible systemic and neuroinflammatory links to AD, including hematogenous dissemination of periodontal microbial products and potential access to the CNS through olfactory pathways, direct evidence for analogous oral–retina or nasal–retina microbiome interactions remains limited. Whether these microbiome compartments can influence retinal inflammation or neurodegeneration through systemic or local pathways therefore warrants further investigation.

## 8. Methodological Challenges in Low-Biomass Microbiome Research

A fundamental challenge in studying the microbiome of the eye, brain, and other low-biomass tissues is distinguishing genuine biological signal from contamination introduced during sampling, DNA extraction, PCR amplification, sequencing, or bioinformatic processing. Unlike the gut, where microbial biomass is high and the signal-to-noise ratio is favorable, the brain and ocular surface contain very low microbial loads [103,104]. Even trace environmental or reagent-derived DNA can dominate sequencing results, as demonstrated by the placental microbiome controversy, a cautionary precedent in which a claimed resident microbiome was subsequently shown to reflect contamination from reagents and the ambient environment [7,105].

The high host DNA-to-bacterial DNA ratio in low-biomass tissues compounds this problem by increasing the risk of off-target amplification, whereby non-bacterial sequences are erroneously assigned to bacterial taxa, generating false-positive findings across many amplicon sequencing pipelines [106]. This phenomenon has been directly documented in brain microbiome studies, where off-target amplification produced apparent bacterial signals in both healthy and Parkinson’s disease individuals that were not reproducible with complementary methods. Rigorous methodological standards for low-biomass microbiome studies should include: (1) extensive negative controls at every experimental step, including sampling blanks, extraction blanks, and no-template PCR controls; (2) quantitative microbial load assessment by qPCR to confirm that signal exceeds background; (3) sterile sampling procedures and replicate sampling; (4) host DNA depletion strategies prior to sequencing; (5) validation using complementary, non-PCR-based methods such as fluorescence in situ hybridization, culture, or immunohistochemistry; and (6) spatially resolved sampling to distinguish surface contamination from true tissue-level detection [107,108]. Table 3 summarizes the contamination control measures employed by key brain microbiome studies and provides an assessment of their overall methodological rigor.

Critically, extensive contamination controls do not by themselves establish the presence of a resident brain microbiome. Studies such as AlzPI [13] and Ko et al. [62] emphasize that viability, anatomical localization, and functional activity must also be demonstrated. These requirements are particularly important for ocular microbiome research because the ocular surface has a low microbial biomass and is continuously exposed to environmental contamination. The same considerations apply with greater force to the ocular surface, where the tissue is continuously exposed to the environment, baseline microbial loads are minimal, and the potential for procedural contamination is high.

The ocular surface presents additional challenges because microbial biomass is low, the surface is exposed to the environment and detected community composition can vary with sampling modality and anatomical site [91,96]. Standardized sampling should incorporate sampling blanks and environmental controls, quantitative microbial-load assessment, and documentation of ocular-specific confounders, including dry eye or other ocular surface disease, contact lens wear, topical antibiotics, ophthalmic medications and preservatives, and recent ocular procedures. These factors are particularly important when distinguishing a reproducible disease-associated signal from contamination or clinically induced alterations in the ocular microbiome.

## 9. Future Directions and Research Framework

Advancing the field of ocular microbiome–AD research requires moving beyond descriptive association studies toward mechanistic experimental designs that can establish causality. The following priorities represent a staged research framework for validating or refuting the ocular microbiome hypothesis in AD:

First: Establish rigorous reference datasets. Longitudinal prospective studies are needed to determine whether ocular microbiome alterations precede or predict AD progression. These studies should include well-characterized participants and collect ocular microbiome profiles together with retinal imaging, tear proteomics and lipidomics, and established AD biomarkers. Integrating these measures will help determine whether ocular microbial signatures provide information beyond established risk factors and biomarkers.

Second: Apply mandatory contamination controls. All future low-biomass ocular and brain microbiome studies must incorporate the full suite of controls detailed above. Transparent reporting of negative control profiles and raw data sharing should be required for publication in this field, as recently recommended by Fierer et al. [108].

Third: Test directionality and causality experimentally. Germ-free and gnotobiotic animal models colonized with specific ocular-derived taxa (*C. acnes*, *Acinetobacter* spp.) should be examined for AD-relevant pathology in retina and brain. Conversely, models of ocular dysbiosis (e.g., following topical antibiotic disruption or model ocular surface disease) should be assessed for retinal and CNS inflammatory consequences.

Fourth: Utilize spatial biology for precise localization. Spatial transcriptomics, spatial metabolomics, and in situ microbial detection could improve the anatomical resolution of ocular–brain studies. Applying these approaches to matched retinal, optic nerve, and brain tissues from the same donors would help determine whether ocular-associated organisms or microbial products can be identified within anatomically connected neural tissues.

Fifth: Evaluate tear fluid as a non-invasive biomarker matrix. Tear proteomics, lipidomics, and microbiome profiling represent minimally invasive approaches for accessing ocular surface biology at scale. The presence of Aβ peptides, cytokines, and microbial-associated molecular patterns in tear fluid warrants systematic investigation as a combined neurodegenerative and dysbiotic biomarker platform.

Sixth: Address sex as a biological variable. Both AD and dry eye disease disproportionately affect women, and estrogen and androgen receptor expression in retinal and CNS cell types modulate neuroinflammatory and neuroprotective pathways in sex-specific ways. All future studies in this area should include sex-stratified analyses.

Beyond hypothesis testing, the ocular microbiome–AD framework has translational potential. Importantly, the potential value of ocular microbiome profiling should not be viewed as a replacement for established retinal imaging or emerging tear-based biomarkers. At present, its extremely low biomass, susceptibility to contamination, and lack of demonstrated association with AD progression substantially limit its diagnostic utility. Rather, its potential value lies in providing complementary information on ocular microbial ecology and host–microbe interactions that is not directly captured by structural retinal imaging or host-derived molecular biomarkers. The accessibility of the ocular surface also permits minimally invasive, repeated sampling. Future longitudinal studies should therefore determine whether ocular microbiome profiling provides incremental diagnostic, prognostic, or mechanistic information when integrated with retinal imaging, tear proteomics and lipidomics, and established AD biomarkers.

**Figure 2:**
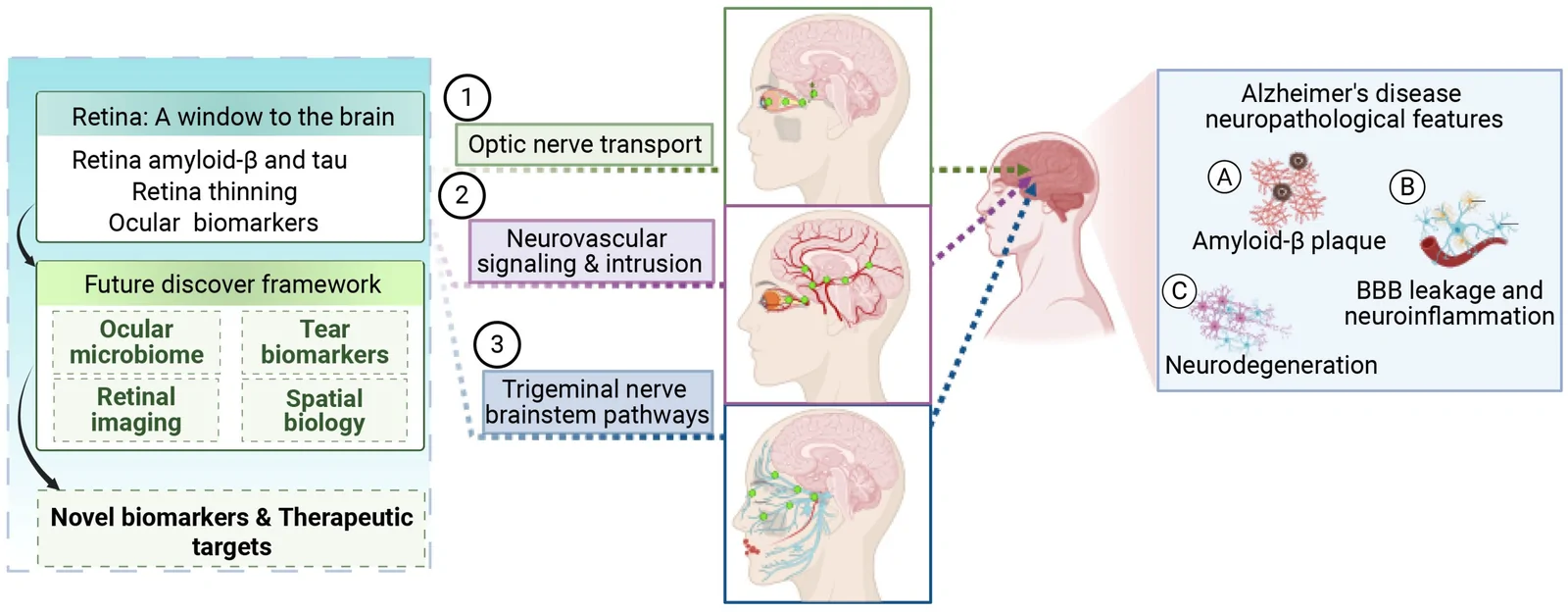
Microbiome compartments implicated in Alzheimer’s disease: emerging biomarkers and therapeutic targets. Schematic overview highlighting microbiome niches associated with Alzheimer’s disease (AD) and their potential contribution to disease pathogenesis. Among these, the ocular microbiome represents a promising and accessible source of biomarkers and therapeutic targets. Ocular-based approaches, including retinal imaging, tear-fluid biomarkers, and spatial biology analyses, may facilitate the investigation of microbiome–host interactions and disease progression. Proposed mechanisms linking the ocular microbiome (green dots) to AD include transport via the optic nerve, neurovascular and blood–brain barrier (BBB) dysfunction, trigeminal nerve- and brainstem-mediated pathways, amyloid-β plaque formation, neurodegeneration, BBB leakage, and neuroinflammation. These pathways may contribute to the characteristic neuropathological features of AD and provide opportunities for future diagnostic and therapeutic interventions. Figure createdusing BioRender.

If a causal contribution of ocular dysbiosis to AD pathogenesis is established, the ocular surface would represent a uniquely accessible therapeutic target (Figure 2). This will warrant future topical antimicrobial or probiotic interventions, tear-replacement formulations, and ocular surface immunomodulation, all of which are clinically deployable strategies that could be rapidly evaluated in proof-of-concept trials.

Clinical translation will also require analytical and regulatory standardization. Specimen collection, storage, assay or sequencing platforms, contamination-control criteria, bioinformatic pipelines and reporting will need harmonization before an ocular microbiome signature can be considered a clinically deployable biomarker. Regulatory evaluation would additionally require robust analytical validity, reproducibility across laboratories and populations, predefined clinical performance criteria and evidence that the assay provides clinically meaningful information beyond currently available AD biomarkers.

## 10. Conclusions

Microbiome research has substantially broadened our understanding of potential contributors to AD beyond the traditional amyloid- and tau-centered frameworks. The strongest current evidence supports associations and biologically plausible contributions of gut and oral microbial dysbiosis in modulating neuroinflammation, Aβ aggregation, and cognitive decline, through systemic inflammatory signaling, barrier disruption, and direct brain-accessible microbial virulence factors. Evidence for nasal and ear microbiome contributions is mechanistically plausible but lacks experimental validation, and evidence for ear microbiome involvement is weakest of all. The ocular microbiome occupies a position of biological interest but current evidential uncertainty. The ocular anatomical proximity to the brain, its shared developmental origins with the CNS, the presence of Aβ and tau pathology in the retinas of AD patients, and the epidemiological parallels between ocular diseases and AD collectively motivate investigation. The identification of *C. acnes* and *Acinetobacter johnsonii* in both ocular specimens and some AD brain studies generates an intriguing but unconfirmed hypothesis. Critically, the detection of shared taxa does not constitute evidence of microbial translocation between the eye and brain. Such observations may instead reflect common environmental exposures, shared ecological niches, or methodological limitations inherent to low-biomass microbiome research. Consequently, these findings require further validation using rigorously controlled studies and well-characterized post-mortem human tissues.

Despite its biological plausibility, the current human evidence linking the ocular microbiome to AD remains limited and is largely cross-sectional and associative. It is therefore unclear whether microbial alterations precede disease onset, contribute to retinal and cerebral pathology, or arise secondary to ageing, neurodegeneration, or environmental factors. Longitudinal studies using standardized sampling, rigorous contamination controls, and integrated retinal, molecular, and AD biomarker assessments are needed to establish temporal relationships. Mechanistic studies will also be essential to determine whether ocular microbial alterations can directly influence retinal inflammation, Aβ accumulation, barrier dysfunction, or other AD-related processes before clinical translation can be considered.

Nevertheless, the ocular microbiome represents an attractive potential target because the ocular surface is readily accessible, can be repeatedly sampled, and may permit localized intervention. If a causal role is established, strategies aimed at restoring microbial homeostasis or reducing pathogenic microbial and inflammatory signals could offer opportunities for targeted therapeutic intervention while potentially minimizing systemic effects. At present, however, the diagnostic and therapeutic potential of targeting the ocular microbiome remains speculative and requires substantial validation through longitudinal and mechanistic studies.

## Figures and Tables

**Table 2:** Non-ocular and ocular microbiome–AD associations graded by evidence category and methodological rigor.

Evidence Category	Microbiome Site	Key Species/Finding	Evidence Strength	Limitations & References
Non-ocular microbiome-related evidence				
Direct brain detection	Nasal/Respiratory	*C. pneumoniae* detected by PCR, nested PCR, RT-PCR, culture, electron microscopy, and IHC in hippocampus, temporal, frontal, parietal, prefrontal cortex and occipital lobe of AD brains. Consistently localized to regions of neuropathology.	Moderate	Limited independent replication across groups; no causal mechanistic proof; detection in FFPE and frozen samples, but contamination controls vary [53,54].
	Oral surface/Skin	*C. acnes* (*P. acnes*) identified as dominant AD-associated taxon by 16S NGS in FFPE and frozen brain tissue. AD brains harbor higher bacterial burden than cognitively normal controls; increase strongly associated with AD rather than age.	Moderate	Low biomass; contamination risk; FFPE amplification artefacts possible; no viability confirmation [16].
	Oral surface/Skin/Environment	*C. acnes* and *A. johnsonii* associated with AD brains (Dirichlet-multinomial model); *A. junii* predominant in controls by full-length 16S rRNA and AlzPI. Microspatial heterogeneity confirmed; edge-vs-core comparison supports non-contamination origin.	Moderate	Shared taxa with ocular surface; environmental ubiquity of both organisms; detection ≠ viability or trafficking [13].
	Oral/Periodontal	*P. gingivalis* LPS detected in post-mortem parietal lobe (area adjacent to lateral ventricle) in AD but not in matched controls by IHC and immunoblot, suggesting periodontal bacterial access to brain.	Moderate	Single anatomical region; LPS detection ≠ viable organism; no direct mechanistic proof of neuroinflammatory consequence in human tissue [55].
	Systemic/Unknown source	*B. burgdorferi* antigen and DNA (16S rDNA, CTP synthase gene) detected in post-mortem hippocampal and frontal lobe of AD patients; co-localizes with amyloid pathology in antibiotic-resistant biofilms. Case report: first direct spirochaete visualization in AD cortex.	Low–Moderate	Single case report origin; biofilm co-localization is associative; contamination controls present but FISH controls needed [56,57].
	Gut-derived	*E. coli* K99 pili protein and LPS levels higher in AD superior temporal gyrus and frontal white matter vs. controls. *E. shigella* increased in stool of amyloidosis patients alongside reduced anti-inflammatory taxa.	Low–Moderate	Off-target PCR amplification documented for gut-derived taxa in brain; no direct translocation demonstrated; reagents screened but cross-contamination possible [58,59].
	Environmental/Systemic	Fungi (*Cryptococcus*, *Candida*, *Alternaria*, *Botrytis*, *Cladosporium*, *Malassezia*, and 14 other genera) detected in frozen brain (frontal, cerebellar, entorhinal cortex, choroidal plexus) by NGS and nested PCR. Multiple *Candida* spp. confirmed by IHC/IB/PCR in separate cohort.	Low–Moderate	Fungi ubiquitous in environment; reagent contamination plausible despite controls; Internal transcribed spacer (ITS) PCR prone to environmental signal; Pisa et al. claim fungi as etiological agent is not supported by design [60,61].
	Unknown/Environmental	*C. normanense* and infection-associated bacteria enriched in AD-associated brain regions (frontal cortex, hippocampus) vs. hypothalamus using 16S rRNA sequencing; 96.6% of reads excluded as off-target during QC.	Low–Moderate	Brain region drives composition more than AD status; rigorous controls (extraction + no-template + decontam/microDecon); biological significance uncertain [62].
Microbial products/Virulence factors	Oral/Periodontal	*A. actinomycetemcomitans* serotype b LPS stimulates hippocampal cells to produce pro-inflammatory cytokines and Aβ in rat model. *P. gingivalis* gingipain proteases modify tau, degrade APOE/LL-37, promote Aβ aggregation.	Moderate	Primarily in vitro and animal models; human translational evidence limited; gingipain data from Dominy et al. [38] not from this table’s sources [63].
Epidemiological association (Serology)	Oral/Systemic multi-pathogen	Higher composite infectious burden (seropositivity to *B. burgdorferi*, *C. pneumoniae*, *H. pylori*) associated with cognitive decline; cumulative microbial exposure index correlates with AD risk.	Moderate	Serological exposure ≠ active CNS infection; confounding by age, immunosenescence, socioeconomic status; cross-sectional design [64].
Epidemiological association (Serology)	Oral/Periodontal	Elevated serum IgG to *P. gingivalis*, *F. nucleatum*, *T. denticola*, *P. intermedia*, *A. actinomycetemcomitans* associated with increased AD risk in case–control serology study.	Moderate	Serology reflects exposure history not active infection; periodontal disease is highly age-prevalent confounding likely [65].
Epidemiological association (Serology/Histology)	Gut/Gastric	*H. pylori* IgG/IgA elevated in AD plasma vs. controls; gastric biopsy & rapid urease test confirms infection association with AD. Higher IgG in vascular dementia than AD suggests non-specific ageing effect.	Low–Moderate	*H. pylori* extremely prevalent in elderly globally; association may reflect age rather than disease-specific mechanism; causality unresolved [66,67].
Epidemiological association (Serology)	Protozoa/Systemic	*T. gondii* IgG seroprevalence significantly higher in AD patients vs. controls in Northern Iranian population.	Low	Single population; *T. gondii* seroprevalence varies widely by geography and diet; no mechanistic data [68].
Bioinformatic inference (Multi-omics/GWAS)	Multiple (Oral, Gut, Viral, Fungal, Protozoan)	BBB proteome enrichment, exosome association, and tissue distribution analysis of AD-associated GWAS genes implicate polymicrobial invasion (*B. burgdorferi*, *C. pneumoniae*, *P. gingivalis*, *H. pylori*, CMV, HSV-1, HERV-W, HIV-1, Epstein–Barr, Hepatitis C, Influenza, *C. albicans*, *C. neoformans*, *T. gondii*, *T. cruzi*).	Weak	Entirely indirect; no experimental validation; GWAS-to-pathogen mapping is speculative; no mechanistic confirmation of actual microbial presence or activity [69].
Ocular microbiome-related evidence				
Circumstantial overlap (Shared taxa—Ocular surface)	Ocular surface	*C. acnes* and *A. johnsonii*, the two taxa most associated with AD brain by AlzPI, are also among the most commonly detected organisms in healthy and diseased ocular surface specimens (conjunctiva, lid margin, Demodex blepharitis samples).	Weak—Circumstantial	Shared taxa do not establish microbial trafficking between eye and brain; both organisms are ubiquitous skin commensals; overlap equally attributable to contamination, age-related ecological convergence, or common environmental source. No direct evidence of ocular-to-brain translocation [13,14,15].

Key: IHC = immunohistochemistry; PCR = polymerase chain reaction; NGS = next-generation sequencing; RT-PCR = reverse transcription PCR; FISH = fluorescence in situ hybridization; LPS = lipopolysaccharide; FFPE = formalin-fixed paraffin-embedded; AlzPI = Alzheimer’s Pathobiome Initiative; BBB = blood–brain barrier; GWAS = genome-wide association study.

**Table 3:** Key brain microbiome studies and their contamination control strategies.

Study	Organism(s)	Method	Key Contamination Controls	Overall Rigour
Thwe et al. (AlzPI) [13]	*C. acnes*, *A. johnsonii*	Full-length 16S rRNA	Sterile biosafety cabinet; edge-vs-core comparison; decontam + microDecon algorithms; extraction blanks	High
Ko et al. [62]	*C. normanense*	16S rRNA sequencing	Extraction + no-template controls; 96.6% off-target reads excluded; histological controls	High
Emery et al. [16]	*C. acnes*	16S NGS	Parallel FFPE/frozen; no-template controls; non-AD controls; blood microbiome comparison	Moderate–High
Gerard et al. [54]	*C. pneumoniae*	Nested PCR, RT-PCR	Water blanks; duplicate assays; sequencing verification	Moderate
Zhan et al. [58]	*E. coli*/*Shigella*	PCR, Western blot	Reagents screened for *E. coli* DNA; age-matched controls	Moderate
Balin et al. [53]	*C. pneumoniae*	PCR, RT-PCR, culture, IHC	Separate labs; no cross-reactivity; negative species controls	Moderate
Pisa et al. [60]	Fungi (*C. albicans*, *Malassezia*)	Nested PCR, IHC	Separate pre/post-PCR areas; aerosol-resistant tips; multiple negative controls; FFPE + frozen comparison	Moderate
Alonso et al. [61]	Fungi (*Cryptococcus*, *Candida*, *Alternaria*)	NGS, nested PCR	Tri-distilled water controls; dedicated glassware; multiple negative controls	Moderate

## Data Availability

No new data were created or analyzed in this study. Data sharing is not applicable to this article.

## References

[1] Alzheimer’s Association (2016). 2016 Alzheimer’s disease facts and figures. Alzheimer’s Dement..

[2] DeTure M.A., Dickson D.W. (2019). The neuropathological diagnosis of Alzheimer’s disease. Mol. Neurodegener..

[3] Karran E., De Strooper B. (2022). The amyloid hypothesis in Alzheimer disease: New insights from new therapeutics. Nat. Rev. Drug Discov..

[4] Hampel H., Hardy J., Blennow K., Chen C., Perry G., Kim S.H., Villemagne V.L., Aisen P., Vendruscolo M., Iwatsubo T. (2021). The Amyloid-beta Pathway in Alzheimer’s Disease. Mol. Psychiatry.

[5] Wu C.K., Fuh J.L. (2025). A 2025 update on treatment strategies for the Alzheimer’s disease spectrum. J. Chin. Med. Assoc..

[6] Whitson H.E., Banks W.A., Diaz M.M., Frost B., Kellis M., Lathe R., Schmader K.E., Spudich S.S., Tanzi R., Garden G. (2024). New approaches for understanding the potential role of microbes in Alzheimer’s disease. Brain Behav. Immun. Health.

[7] De Goffau M.C., Lager S., Sovio U., Gaccioli F., Cook E., Peacock S.J., Parkhill J., Charnock-Jones D.S., Smith G.C. (2019). Human placenta has no microbiome but can contain potential pathogens. Nature.

[8] Abelson M.B., Lane K., Slocum C. (2015). The Secrets of Ocular Microbiomes. Review of Ophthalmology Online. http://www.reviewofophthalmology.com/article/the-secrets-of-ocular-microbiomes.

[9] Bathini P., Brai E., Balin B.J., Bimler L., Corry D.B., Devanand D.P., Doty R.L., Ehrlich G.D., Eimer W.A., Fulop T. (2024). Sensory Dysfunction, Microbial Infections, and Host Responses in Alzheimer’s Disease. J. Infect. Dis..

[10] Gomes J.Á.P., Frizon L., Demeda V.F. (2020). Ocular Surface Microbiome in Health and Disease. Asia-Pac. J. Ophthalmol..

[11] Gaire B.P., Koronyo Y., Fuchs D.T., Shi H., Rentsendorj A., Danziger R., Vit J.P., Mirzaei N., Doustar J., Sheyn J. (2024). Alzheimer’s disease pathophysiology in the Retina. Prog. Retin. Eye Res..

[12] London A., Benhar I., Schwartz M. (2013). The retina as a window to the brain-from eye research to CNS disorders. Nat. Rev. Neurol..

[13] Thwe M.N., Mone Y., Sen B., Czerski S., Azad A., Earl J.P., Hall D.C., Ehrlich G.D. (2025). Microspatial Heterogeneities and the Absence of Postmortem Contamination in Alzheimer’s Disease Brain Microbiota: An Alzheimer’s Pathobiome Initiative (AlzPI) Study. Microorganisms.

[14] Fu Y., Wu J., Wang D., Li T., Shi X., Li L., Zhu M., Zhang Z., Yu X., Dai Q. (2022). Metagenomic profiling of ocular surface microbiome changes in Demodex blepharitis patients. Front. Cell. Infect. Microbiol..

[15] Schlegel I., De Goüyon Matignon de Pontourade C.M., Lincke J.-B., Keller I., Zinkernagel M.S., Zysset-Burri D.C. (2023). The human ocular surface microbiome and its associations with the tear proteome in dry eye disease. Int. J. Mol. Sci..

[16] Emery D.C., Shoemark D.K., Batstone T.E., Waterfall C.M., Coghill J.A., Cerajewska T.L., Davies M., West N.X., Allen S.J. (2017). 16S rRNA next generation sequencing analysis shows bacteria in Alzheimer’s post-mortem brain. Front. Aging Neurosci..

[17] Butovsky O., Rosenzweig N. (2025). Alzheimer’s disease and age-related macular degeneration: Shared and distinct immune mechanisms. Immunity.

[18] Zhang L., Liang B., Li M. (2025). Cross-Talk Between Glaucoma and Alzheimer’s Disease: S100A11 as a Dual Regulator of Neurodegeneration Through Inflammation and Antioxidant Signaling. FASEB J..

[19] Zemaitiene R., Gorgadze G., Mockaitiene L. (2025). Ocular Surface Changes Associated with Neurological Diseases. Medicina.

[20] Peng M., Zeng Q., Zheng W., Xia X. (2025). Peripheral Choroid/RPE/Sclera as a Shared Pathogenic Hub: Multi-Tissue Transcriptomic Profiling Identifies Common Differentially Expressed Genes in Age-Related Macular Degeneration and Alzheimer’s Disease. Mol. Neurobiol..

[21] Kim W.S., Halliday G.M. (2026). Astrocytic lipid dysregulation as an early driver of neurodegeneration. Nat. Rev. Neurol..

[22] Readhead B., Haure-Mirande J.V., Funk C.C., Richards M.A., Shannon P., Haroutunian V., Sano M., Liang W.S., Beckmann N.D., Price N.D. (2018). Multiscale Analysis of Independent Alzheimer’s Cohorts Finds Disruption of Molecular, Genetic, and Clinical Networks by Human Herpesvirus. Neuron.

[23] Cullell N., Caruana G., Elias-Mas A., Delgado-Sanchez A., Artero C., Buongiorno M.T., Almeria M., Ray N.J., Correa S.A.L., Krupinski J. (2025). Glymphatic system clearance and Alzheimer’s disease risk: A CSF proteome-wide study. Alzheimer’s Res. Ther..

[24] Gaire B.P., Koronyo Y., Vit J.-P., Hutton A., Subedi L., Fuchs D.-T., Swerdlow N., Rentsendorj A., Shahin S., Martinon D. (2026). Identification of Chlamydia pneumoniae and NLRP3 inflammasome activation in Alzheimer’s disease retina. Nat. Commun..

[25] Leng F., Edison P. (2021). Neuroinflammation and microglial activation in Alzheimer disease: Where do we go from here?. Nat. Rev. Neurol..

[26] Hardy J.A., Higgins G.A. (1992). Alzheimer’s disease: The amyloid cascade hypothesis. Science.

[27] Selkoe D.J., Hardy J. (2016). The amyloid hypothesis of Alzheimer’s disease at 25 years. EMBO Mol. Med..

[28] Liu X., Liu Y., Ji S. (2021). Secretases Related to Amyloid Precursor Protein Processing. Membranes.

[29] Zhang B., Yu X., Zhang X. (2026). The evolving landscape of amyloid-targeting therapies in Alzheimer’s disease: Progress and challenges. Neurol. Sci..

[30] Granzotto A., Sensi S.L. (2024). Once upon a time, the Amyloid Cascade Hypothesis. Ageing Res. Rev..

[31] Kang J., Lemaire H.G., Unterbeck A., Salbaum J.M., Masters C.L., Grzeschik K.H., Multhaup G., Beyreuther K., Muller-Hill B. (1987). The precursor of Alzheimer’s disease amyloid A4 protein resembles a cell-surface receptor. Nature.

[32] Koronyo-Hamaoui M., Koronyo Y., Ljubimov A.V., Miller C.A., Ko M.K., Black K.L., Schwartz M., Farkas D.L. (2011). Identification of amyloid plaques in retinas from Alzheimer’s patients and noninvasive in vivo optical imaging of retinal plaques in a mouse model. Neuroimage.

[33] Zhang J., Kong G., Yang J., Pang L., Li X. (2025). Pathological mechanisms and treatment progression of Alzheimer’s disease. Eur. J. Med. Res..

[34] Kametani F., Hasegawa M. (2018). Reconsideration of Amyloid Hypothesis and Tau Hypothesis in Alzheimer’s Disease. Front. Neurosci..

[35] Schon C., Hoffmann N.A., Ochs S.M., Burgold S., Filser S., Steinbach S., Seeliger M.W., Arzberger T., Goedert M., Kretzschmar H.A. (2012). Long-term in vivo imaging of fibrillar tau in the retina of P301S transgenic mice. PLoS ONE.

[36] Bostanciklioglu M. (2019). The role of gut microbiota in pathogenesis of Alzheimer’s disease. J. Appl. Microbiol..

[37] Barron A.E., Lin J.S., Ryder M.I., Bergman P. (2026). The dysregulation of innate immunity by *Porphyromonas gingivalis* in the etiology of Alzheimer’s disease. J. Intern. Med..

[38] Dominy S.S., Lynch C., Ermini F., Benedyk M., Marczyk A., Konradi A., Nguyen M., Haditsch U., Raha D., Griffin C. (2019). *Porphyromonas gingivalis* in Alzheimer’s disease brains: Evidence for disease causation and treatment with small-molecule inhibitors. Sci. Adv..

[39] Brown G.C., Heneka M.T. (2024). The endotoxin hypothesis of Alzheimer’s disease. Mol. Neurodegener..

[40] Cani P.D., Amar J., Iglesias M.A., Poggi M., Knauf C., Bastelica D., Neyrinck A.M., Fava F., Tuohy K.M., Chabo C. (2007). Metabolic endotoxemia initiates obesity and insulin resistance. Diabetes.

[41] Mehta R.I., Mehta R.I. (2023). The Vascular-Immune Hypothesis of Alzheimer’s Disease. Biomedicines.

[42] Korte N., Nortley R., Attwell D. (2020). Cerebral blood flow decrease as an early pathological mechanism in Alzheimer’s disease. Acta Neuropathol..

[43] Wostyn P. (2021). The “ocular glymphatic clearance system”: A key missing piece of the Alzheimer’s disease-glaucoma puzzle found?. Eye.

[44] Wang X., Lou N., Eberhardt A., Yang Y., Kusk P., Xu Q., Forstera B., Peng S., Shi M., Ladron-de-Guevara A. (2020). An ocular glymphatic clearance system removes beta-amyloid from the rodent eye. Sci. Transl. Med..

[45] Nedergaard M., Goldman S.A. (2020). Glymphatic failure as a final common pathway to dementia. Science.

[46] D’Alessandro M.C.B., Kanaan S., Geller M., Pratico D., Daher J.P.L. (2025). Mitochondrial dysfunction in Alzheimer’s disease. Ageing Res. Rev..

[47] Reutzel M., Grewal R., Joppe A., Eckert G.P. (2022). Age-Dependent Alterations of Cognition, Mitochondrial Function, and Beta-Amyloid Deposition in a Murine Model of Alzheimer’s Disease—A Longitudinal Study. Front. Aging Neurosci..

[48] Scarano N., Musumeci F., Casini B., Brullo C., D’Ursi P., Fossa P., Schenone S., Cichero E. (2025). Alzheimer’s Disease Etiology Hypotheses and Therapeutic Strategies: A Perspective. Int. J. Mol. Sci..

[49] Sweeney M.D., Sagare A.P., Zlokovic B.V. (2018). Blood-brain barrier breakdown in Alzheimer disease and other neurodegenerative disorders. Nat. Rev. Neurol..

[50] Phan K., He Y., Fu Y., Dzamko N., Bhatia S., Gold J., Rowe D., Ke Y.D., Ittner L.M., Hodges J.R. (2021). Pathological manifestation of human endogenous retrovirus K in frontotemporal dementia. Commun. Med..

[51] Kern L., Mastandrea I., Melekhova A., Elinav E. (2025). Mechanisms by which microbiome-derived metabolites exert their impacts on neurodegeneration. Cell Chem. Biol..

[52] Teng M., Ma R., Liu S., Zhang G., Zou J., Fu Q. (2025). Bacterial infection hypothesis-based therapies for Alzheimer’s disease. Chem. Eng. J..

[53] Balin B.J., Gerard H.C., Arking E.J., Appelt D.M., Branigan P.J., Abrams J.T., Whittum-Hudson J.A., Hudson A.P. (1998). Identification and localization of Chlamydia pneumoniae in the Alzheimer’s brain. Med. Microbiol. Immunol..

[54] Gerard H.C., Dreses-Werringloer U., Wildt K.S., Deka S., Oszust C., Balin B.J., Frey W.H., Bordayo E.Z., Whittum-Hudson J.A., Hudson A.P. (2006). *Chlamydophila* (*Chlamydia*) *pneumoniae* in the Alzheimer’s brain. FEMS Immunol. Med. Microbiol..

[55] Poole S., Singhrao S.K., Kesavalu L., Curtis M.A., Crean S. (2013). Determining the presence of periodontopathic virulence factors in short-term postmortem Alzheimer’s disease brain tissue. J. Alzheimer’s Dis..

[56] MacDonald A.B., Miranda J.M. (1987). Concurrent neocortical borreliosis and Alzheimer’s disease. Hum. Pathol..

[57] Senejani A.G., Maghsoudlou J., El-Zohiry D., Gaur G., Wawrzeniak K., Caravaglia C., Khatri V.A., MacDonald A., Sapi E. (2022). Borrelia burgdorferi co-localizing with amyloid markers in Alzheimer’s disease brain tissues. J. Alzheimer’s Dis..

[58] Zhan X., Stamova B., Jin L.-W., DeCarli C., Phinney B., Sharp F.R. (2016). Gram-negative bacterial molecules associate with Alzheimer disease pathology. Neurology.

[59] Cattaneo A., Cattane N., Galluzzi S., Provasi S., Lopizzo N., Festari C., Ferrari C., Guerra U.P., Paghera B., Muscio C. (2017). Association of brain amyloidosis with pro-inflammatory gut bacterial taxa and peripheral inflammation markers in cognitively impaired elderly. Neurobiol. Aging.

[60] Pisa D., Alonso R., Rabano A., Rodal I., Carrasco L. (2015). Different Brain Regions are Infected with Fungi in Alzheimer’s Disease. Sci. Rep..

[61] Alonso R., Pisa D., Aguado B., Carrasco L. (2017). Identification of Fungal Species in Brain Tissue from Alzheimer’s Disease by Next-Generation Sequencing. J. Alzheimer’s Dis..

[62] Ko Y.K., Kim E., Lee E.J., Nam S.J., Kim Y., Kim S., Choi S.Y., Kim H.Y., Choi Y. (2024). Enrichment of infection-associated bacteria in the low biomass brain bacteriota of Alzheimer’s disease patients. PLoS ONE.

[63] Diaz-Zuniga J., Munoz Y., Melgar-Rodriguez S., More J., Bruna B., Lobos P., Monasterio G., Vernal R., Paula-Lima A. (2019). Serotype b of *Aggregatibacter actinomycetemcomitans* triggers pro-inflammatory responses and amyloid beta secretion in hippocampal cells: A novel link between periodontitis and Alzheimer s disease?. J. Oral Microbiol..

[64] Bu X.L., Yao X.Q., Jiao S.S., Zeng F., Liu Y.H., Xiang Y., Liang C.R., Wang Q.H., Wang X., Cao H.Y. (2015). A study on the association between infectious burden and Alzheimer’s disease. Eur. J. Neurol..

[65] Stein P.S., Steffen M.J., Smith C., Jicha G., Ebersole J.L., Abner E., Dawson D. (2012). Serum antibodies to periodontal pathogens are a risk factor for Alzheimer’s disease. Alzheimer’s Dement..

[66] Malaguarnera M., Bella R., Alagona G., Ferri R., Carnemolla A., Pennisi G. (2004). Helicobacter pylori and Alzheimer’s disease: A possible link. Eur. J. Intern. Med..

[67] Kountouras J., Tsolaki M., Gavalas E., Boziki M., Zavos C., Karatzoglou P., Chatzopoulos D., Venizelos I. (2006). Relationship between Helicobacter pylori infection and Alzheimer disease. Neurology.

[68] Rostami F., Hashemi S.A., Shaghaghi B., Fazaeli M., Rezaei M., Pagheh A.S., Arghavan M.S., Pazoki H., Rezaei F. (2026). Toxoplasma gondii infection and Alzheimer’s disease: A case-control study in a Northern Iranian population. J. Parasit. Dis..

[69] Carter C.J. (2017). Genetic, transcriptome, proteomic, and epidemiological evidence for blood-brain barrier disruption and polymicrobial brain invasion as determinant factors in Alzheimer’s disease. J. Alzheimer’s Dis. Rep..

[70] Sender R., Fuchs S., Milo R. (2016). Revised Estimates for the Number of Human and Bacteria Cells in the Body. PLoS Biol..

[71] Eckburg P.B., Bik E.M., Bernstein C.N., Purdom E., Dethlefsen L., Sargent M., Gill S.R., Nelson K.E., Relman D.A. (2005). Diversity of the human intestinal microbial flora. Science.

[72] McCallum G., Tropini C. (2024). The gut microbiota and its biogeography. Nat. Rev. Microbiol..

[73] Kobayashi Y., Sugahara H., Shimada K., Mitsuyama E., Kuhara T., Yasuoka A., Kondo T., Abe K., Xiao J.Z. (2017). Therapeutic potential of Bifidobacterium breve strain A1 for preventing cognitive impairment in Alzheimer’s disease. Sci. Rep..

[74] Kang J.W., Khatib L.A., Heston M.B., Dilmore A.H., Labus J.S., Deming Y., Schimmel L., Blach C., McDonald D., Gonzalez A. (2025). Gut microbiome compositional and functional features associate with Alzheimer’s disease pathology. Alzheimer’s Dement..

[75] Peng S., Li J.J., Song W., Li Y., Zeng L., Liang Q., Wen X., Shang H., Liu K., Peng P. (2024). CRB1-associated retinal degeneration is dependent on bacterial translocation from the gut. Cell.

[76] Parker A., Romano S., Ansorge R., Aboelnour A., Le Gall G., Savva G.M., Pontifex M.G., Telatin A., Baker D., Jones E. (2022). Fecal microbiota transfer between young and aged mice reverses hallmarks of the aging gut, eye, and brain. Microbiome.

[77] Escapa I.F., Chen T., Huang Y., Gajare P., Dewhirst F.E., Lemon K.P. (2018). New Insights into Human Nostril Microbiome from the Expanded Human Oral Microbiome Database (eHOMD): A Resource for the Microbiome of the Human Aerodigestive Tract. mSystems.

[78] Baker J.L., Mark Welch J.L., Kauffman K.M., McLean J.S., He X. (2024). The oral microbiome: Diversity, biogeography and human health. Nat. Rev. Microbiol..

[79] Eke P.I., Wei L., Borgnakke W.S., Thornton-Evans G., Zhang X., Lu H., McGuire L.C., Genco R.J. (2016). Periodontitis prevalence in adults ≥ 65 years of age, in the USA. Periodontol. 2000.

[80] Huang Q., Dong X. (2022). Prevalence of periodontal disease in middle-aged and elderly patients and its influencing factors. Am. J. Transl. Res..

[81] Dioguardi M., Crincoli V., Laino L., Alovisi M., Sovereto D., Mastrangelo F., Russo L.L., Muzio L.L. (2020). The Role of Periodontitis and Periodontal Bacteria in the Onset and Progression of Alzheimer’s Disease: A Systematic Review. J. Clin. Med..

[82] Rozenblum G., Ait-Aissa K., Zahran G., Alipour M., Sahyoun A.M., Munkhsaikhan U., Kassan A., Ishrat T., Wang Q., Abidi A.H. (2025). Unraveling the oral microbiome’s role in Alzheimer’s disease: From pathophysiology to therapeutic potential. Alzheimer’s Dement..

[83] Roghmann M.C., Lydecker A.D., Hittle L., DeBoy R.T., Nowak R.G., Johnson J.K., Mongodin E.F. (2017). Comparison of the Microbiota of Older Adults Living in Nursing Homes and the Community. mSphere.

[84] Whelan F.J., Verschoor C.P., Stearns J.C., Rossi L., Luinstra K., Loeb M., Smieja M., Johnstone J., Surette M.G., Bowdish D.M. (2014). The loss of topography in the microbial communities of the upper respiratory tract in the elderly. Ann. Am. Thorac. Soc..

[85] Hong S., Baek S.H., Lai M.K.P., Arumugam T.V., Jo D.G. (2024). Aging-associated sensory decline and Alzheimer’s disease. Mol. Neurodegener..

[86] Devanand D. (2016). Olfactory identification deficits, cognitive decline, and dementia in older adults. Am. J. Geriatr. Psychiatry.

[87] Song H., Zou J., Sun Z., Pu Y., Qi W., Sun L., Li Q., Yuan C., Wang X., Gao X. (2025). Nasal microbiome in relation to olfactory dysfunction and cognitive decline in older adults. Transl. Psychiatry.

[88] Little C.S., Hammond C.J., MacIntyre A., Balin B.J., Appelt D.M. (2004). Chlamydia pneumoniae induces Alzheimer-like amyloid plaques in brains of BALB/c mice. Neurobiol. Aging.

[89] Xie J., Tian S., Liu J., Cao R., Yue P., Cai X., Shang Q., Yang M., Han L., Zhang D.-K. (2022). Dual role of the nasal microbiota in neurological diseases—An unignorable risk factor or a potential therapy carrier. Pharmacol. Res..

[90] Burton M., Krumbeck J.A., Wu G., Tang S., Prem A., Gupta A.K., Dawson T.L. (2022). The adult microbiome of healthy and otitis patients: Definition of the core healthy and diseased ear microbiomes. PLoS ONE.

[91] Ozkan J., Willcox M., Wemheuer B., Wilcsek G., Coroneo M., Thomas T. (2019). Biogeography of the human ocular microbiota. Ocul. Surf..

[92] Lee S.H., Oh D.H., Jung J.Y., Kim J.C., Jeon C.O. (2012). Comparative ocular microbial communities in humans with and without blepharitis. Investig. Ophthalmol. Vis. Sci..

[93] Suzuki T., Sutani T., Nakai H., Shirahige K., Kinoshita S. (2020). The microbiome of the meibum and ocular surface in healthy subjects. Investig. Ophthalmol. Vis. Sci..

[94] Ozkan J., Nielsen S., Diez-Vives C., Coroneo M., Thomas T., Willcox M. (2017). Temporal Stability and Composition of the Ocular Surface Microbiome. Sci. Rep..

[95] Andersson J., Vogt J.K., Dalgaard M.D., Pedersen O., Holmgaard K., Heegaard S. (2021). Ocular surface microbiota in patients with aqueous tear-deficient dry eye. Ocul. Surf..

[96] Katzka W., Dong T.S., Luu K., Lagishetty V., Sedighian F., Arias-Jayo N., Jacobs J.P., Hsu H.Y. (2021). The Ocular Microbiome Is Altered by Sampling Modality and Age. Transl. Vis. Sci. Technol..

[97] Patton N., Aslam T., MacGillivray T., Pattie A., Deary I.J., Dhillon B. (2005). Retinal vascular image analysis as a potential screening tool for cerebrovascular disease: A rationale based on homology between cerebral and retinal microvasculatures. J. Anat..

[98] Zhao B., Li Y., Fan Z., Wu Z., Shu J., Yang X., Yang Y., Wang X., Li B., Wang X. (2024). Eye-brain connections revealed by multimodal retinal and brain imaging genetics. Nat. Commun..

[99] Perez V.L., Chen W., Craig J.P., Dogru M., Jones L., Stapleton F., Wolffsohn J.S., Sullivan D.A. (2026). TFOS DEWS III: Executive Summary. Am. J. Ophthalmol..

[100] Romaus-Sanjurjo D., Regueiro U., Lopez-Lopez M., Vazquez-Vazquez L., Ouro A., Lema I., Sobrino T. (2022). Alzheimer’s Disease Seen through the Eye: Ocular Alterations and Neurodegeneration. Int. J. Mol. Sci..

[101] Liu C., Fang Z., He Z., Mao Y., Yang H. (2026). Eye-brain axis: The crosstalk between neurons and immune cells. Eye Discov..

[102] Aliashrafi M., Nasehi M., Zarrindast M.-R., Joghataei M.-T., Zali H., Siadat S.D. (2024). Intracerebroventricular cutibacterium acnes generates manifestations of Alzheimer’s disease-like pathology in the rat hippocampus. Neuroscience.

[103] Ozkan J., Willcox M.D. (2019). The ocular microbiome: Molecular characterisation of a unique and low microbial environment. Curr. Eye Res..

[104] Tang G., Carr A.V., Perez C., Sarmiento K.R., Levy L., Lampe J.W., Diener C., Gibbons S.M. (2025). Metagenomic estimation of absolute bacterial biomass in the mammalian gut through host-derived read normalization. mSystems.

[105] Lauder A.P., Roche A.M., Sherrill-Mix S., Bailey A., Laughlin A.L., Bittinger K., Leite R., Elovitz M.A., Parry S., Bushman F.D. (2016). Comparison of placenta samples with contamination controls does not provide evidence for a distinct placenta microbiota. Microbiome.

[106] Bedarf J.R., Beraza N., Khazneh H., Özkurt E., Baker D., Borger V., Wüllner U., Hildebrand F. (2021). Much ado about nothing? Off-target amplification can lead to false-positive bacterial brain microbiome detection in healthy and Parkinson’s disease individuals. Microbiome.

[107] Eisenhofer R., Minich J.J., Marotz C., Cooper A., Knight R., Weyrich L.S. (2019). Contamination in low microbial biomass microbiome studies: Issues and recommendations. Trends Microbiol..

[108] Fierer N., Leung P.M., Lappan R., Eisenhofer R., Ricci F., Holland S.I., Dragone N., Blackall L.L., Dong X., Dorador C. (2025). Guidelines for preventing and reporting contamination in low-biomass microbiome studies. Nat. Microbiol..

